# Ferroptosis: A New Mechanism in Diabetic Cardiomyopathy

**DOI:** 10.7150/ijms.88476

**Published:** 2024-01-21

**Authors:** Zichong Song, Jingyi Wang, Lijun Zhang

**Affiliations:** 1 Department of Geriatrics, Renmin Hospital of Wuhan University, Wuhan 430060, China.; 2 Department of Neurology, Tongji Hospital, Tongji Medical College, Huazhong University of Science and Technology, Wuhan 430000, China.

**Keywords:** Diabetic cardiomyopathy, Ferroptosis, Mechanism

## Abstract

Diabetic cardiomyopathy (DC) is a pathophysiologic condition caused by diabetes mellitus (DM) in the absence of coronary artery disease, valvular heart disease, and hypertension that can lead to heart failure (HF), manifesting itself in the early stages with left ventricular hypertrophy and diastolic dysfunction, with marked HF and decreased systolic function in the later stages. There is still a lack of direct evidence to prove the exact existence of DC. Ferroptosis is a novel form of cell death characterized by reactive oxygen species (ROS) accumulation and lipid peroxidation. Several cell and animal studies have shown that ferroptosis is closely related to DC progression. This review systematically summarizes the related pathogenic mechanisms of ferroptosis in DC, including the reduction of cardiac RDH10 induced ferroptosis in DC cardiomyocytes which mediated by retinol metabolism disorders; CD36 overexpression caused lipid deposition and decreased GPX4 expression in DC cardiomyocytes, leading to the development of ferroptosis; Nrf2 mediated iron overload and lipid peroxidation in DC cardiomyocytes and promoted ferroptosis; lncRNA-ZFAS1 as a ceRNA, combined with miR-150-5p to inhibit CCND2 expression in DC cardiomyocytes, thereby triggering ferroptosis.

## Introduction

Cell death is one of the most fundamental physiological processes involved in living, marking the end of cell life. Currently, cell death is usually classified as regulated cell death (RCD) and accidental cell death (ACD)^1^. Different from ACD (a biological uncontrolled passive process), RCD refers to an active process controlled by genetical encoded mechanisms. Apoptosis is a classical form of RCD, and an increasingly number of other forms of RCD (such as ferroptosis, necroptosis, parthanatos, oxeiptosis, pyroptosis, and alkaliptosis) have also been discovered^1^. Of these, ferroptosis is an iron-dependent non-apoptotic cell death accompanied by the accumulation of lipid peroxidation, which is different from other types of cell death. As a newly revealed RCD, ferroptosis has been extensively studied in tumors, neurological diseases and cardiovascular diseases^2^-^4^.

Diabetes mellitus (DM) is a metabolic disease featured by hyperglycemia. DM can lead to various types of complications, among which diabetic cardiomyopathy (DC) is one of the most frequent complications and the main cause of heart failure (HF) and death in DM patients^5^-^7^. DC is a kind of myocardial damage characterized by interstitial fibrosis and diastolic dysfunction resulting from DM^8^, ^9^. Its pathogenesis is complex, including hyperglycemia, insulin resistance, oxidative stress, endoplasmic reticulum stress, inflammation, lipid deposition, and elevated levels of advanced glycation end products (AGEs)^10^-^12^. Although DC has been extensively studied in recent decades, understanding of its pathogenesis, diagnostic criteria remains limited, and the presence of DC in patients is still controversial. According to several recent reports, ferroptosis has been implicated in the pathogenesis of DC^13^, ^14^. In order to further understand the pathogenesis of DC, this review takes ferroptosis as a novel research idea of DC pathogenesis, and explores the role of ferroptosis in DC.

## Hallmarks of Ferroptosis

Ferroptosis was first proposed by Dixon et al. in 2012, and is obviously distinguished from other types of cell death through a series of cell morphology, functions and regulatory pathways^15^, ^16^. In terms of biochemistry, the major characteristics of ferroptosis are iron overload and lipid peroxidation. Iron homeostasis is essential to body because iron is a redox-active element involved in many biological processes, such as oxidative phosphorylation, erythropoiesis, DNA synthesis and mitochondrial function^17^. When iron is overloaded in cells, excessive iron can oxidize lipids to increase the levels of intracellular reactive oxygen species (ROS) via the Fenton reaction, promote lipid peroxidation and aggravate ferroptosis. In addition, the consumption of intracellular glutathione (GSH) and the reduction of glutathione peroxidase 4 (GPX4) activity can lead to the accumulation of lipid peroxides, thereby promoting the occurrence of ferroptosis^18^. In terms of morphology, ferroptotic cells show necrosis-like changes, such as cellular swelling, plasma membrane rupture, mitochondrial shrinkage and reduction in number, increased density of lipid bilayers, destruction of mitochondrial cristae and mitochondrial outer membrane^19^. In genetics, cells undergoing ferroptosis occur gene expression changes that regulate iron metabolism and lipid peroxidation. Some overexpressed genes such as Acyl-CoA synthetase long-chain family member 4 (ACSL4) have become biomarkers of ferroptosis^20^.

## The Main Regulatory Mechanisms of Ferroptosis

### Iron Metabolism in Ferroptosis

**Figure 1:**
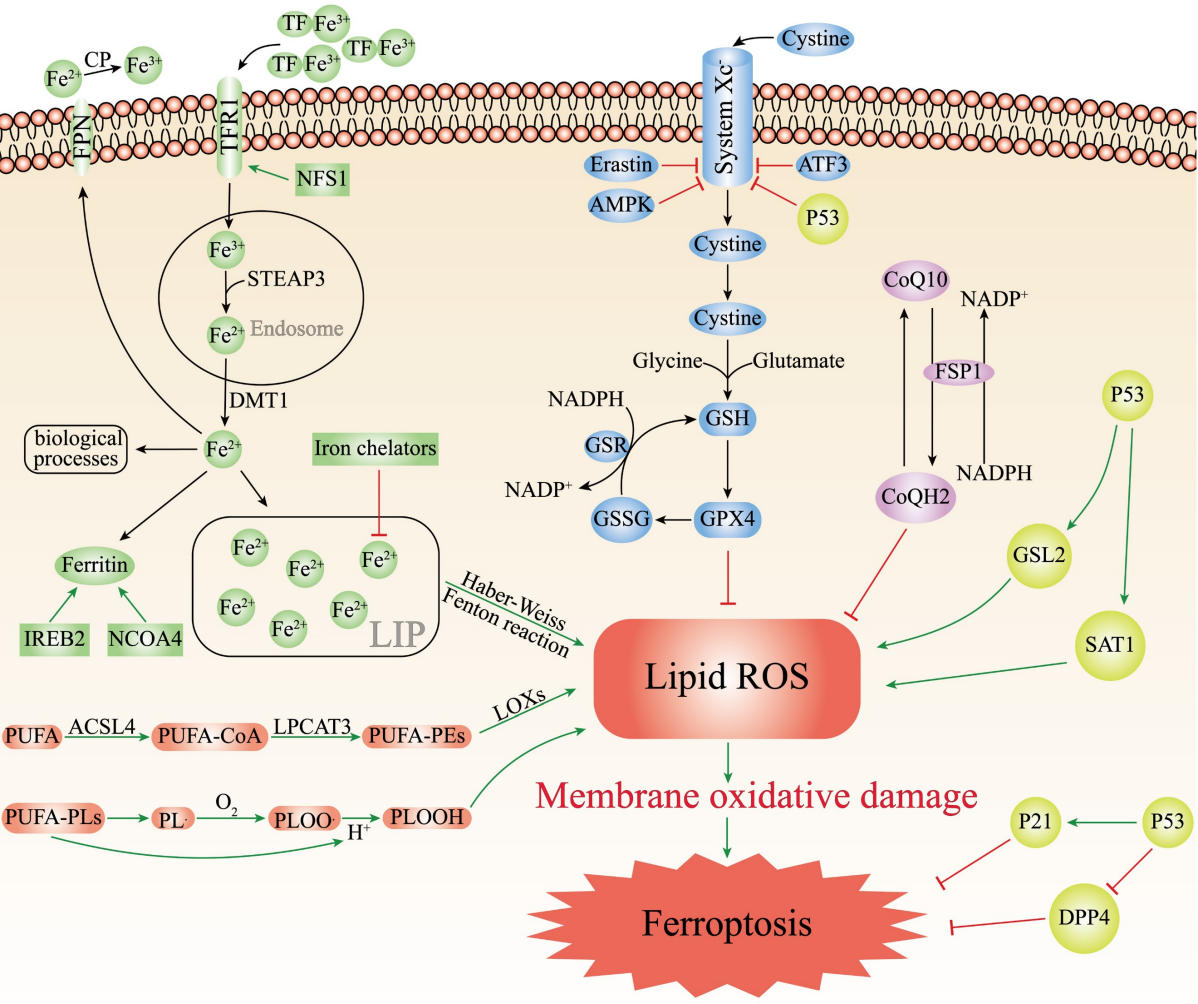
** The main mechanisms of ferroptosis.** The figure shows 5 main pathways of ferroptosis. First, the increased iron uptake and the regulation of NCOA4 and IREB2 can lead to iron overload which causes the production of ROS, leading to ferroptosis; Second, GPX4/GSH/System Xc^-^ pathway is a key pathway for ferroptosis, and inhibition of System Xc^-^, GSH, or GPX4 can lead to ferroptosis; Third, PUFAs can cause ferroptosis by lipid peroxidation through enzymatic and non-enzymatic reactions; Fourth, the FSP1/CoQ10/NAD(P)H parallels the GSH/GPX4, the inhibition of which leads to ferroptosis; Fifth, P53 plays a bidirectional role in different cells. P53 can mediate ferroptosis through enhancing the expression of SAT1 and GSL2 and inhibiting the System Xc^-^. P53 can also restrain ferroptosis either by directly depressing DPP4 activity or by mediating p21 expression.

Iron is an essential trace element required by all living organisms, while inappropriately low or high levels iron content in the body are harmful and can lead to various of diseases^21^. The body needs to replenish approximately 1-2 mg iron from external foods every day^22^. There are two forms of iron in the body: ferric iron (Fe^3+^) and ferrous iron (Fe^2+^). When foods reach the proximal small intestine, cytochrome b can reduce Fe^3+^ to Fe^2+^, which are then absorbed by intestinal epithelial cells (IEC) with the help of the divalent metal transporter 1 (DMT1)^23^. Ferroportin (FPN) can transport Fe^2+^ into the blood circulation participating in the regulation of iron homeostasis^24^. Exported Fe^2+^ will be oxidized to Fe^3+^ through ceruloplasmin (CP), and bound to transferrin (TF) to start the iron cycle^25^. Subsequently, TF-Fe^3+^ binds to transferrin receptor 1 (TFR1) on the surface of cell membrane, forming the TF/TFR1 complex enters the cell endosome through endocytosis^26^. Fe^3+^ will be free and instantly reduced to Fe^2+^ through six-transmembrane epithelial antigen of prostate 3 (STEAP3). After Fe^2+^ leaves the endosome and enters the cytoplasm through DMT1, Fe^2+^ can either be utilized by various biological processes, preserved in the ferritin or made up the labile iron pool (LIP)^27^. Finally, excess iron is transported outside the cell by ferroportin to continue to participate in the iron cycle (Figure 1).

Physiologically, iron homeostasis is properly maintained by complex cascade processes. Pathologically, iron overload can easily generate hydroxyl radicals (·OH) by participating in Fenton and Haber-Weiss reactions, which can contribute to intracellular ROS accumulation and induce ferroptosis^28^. Moreover, the accumulation of ROS can lead to oxidative stress and produce large amounts of superoxide and peroxide, which triggering the release of Fe^2+^ from various iron-containing substances such as ferritin, iron-sulfur clusters and heme, aggravating iron overload^29^, and this will continue to increase oxidative stress by the Fenton reaction and create a vicious cycle. Recently, many studies have indicated that lots of iron-related substances can regulate ferroptosis. Iron chelators (such as deferasirox, dexrazoxane, deferoxamine) can decrease active iron in LIP and suppress ferroptosis^30^. Silencing TFRC that encodes TFR1 can inhibit erastin-induced ferroptosis^31^. Nuclear receptor coactivator 4 (NCOA4) regulates ferroptosis by controlling the autophagic degradation of ferritin^32^. Nitrogen fxation 1 (NFS1) is an iron-sulfur cluster biosynthetic enzyme which can induce the expression of TFR1 and increase the degree of ferroptosis^33^. As the major transcription factor of iron metabolism, iron-responsive element-binding protein 2 (IREB2) can combine with the RNA stem-loop structures in the 3′-untranslated region of mRNA and stabilize transcripts of TFRC or DMT1, thus increasing cell iron concentration^34^. Therefore, silencing IREB2 can reduce sensitivity to ferroptosis (Figure 1).

### Lipid Peroxidation in Ferroptosis

Unrestricted lipid peroxidation is the marker of ferroptosis^35^. Only polyunsaturated fatty acids (PUFAs) are susceptible to peroxidation because they contain specific carbon atoms, and the peroxyl groups can easily replace the attached hydrogen atoms. Lipid peroxidation can be achieved through two pathways, enzymatic or non-enzymatic reactions^36^. In the enzyme-dependent lipid peroxidation, ACSL4 can acylate PUFAs to generate PUFA-CoA, which binds to membrane phospholipids and was catalyzed by lysophosphatidylcholine acyltransferase 3 (LPCAT3) to generate PUFA-containing membrane phospholipids (PUFA-PEs). Eventually, PUFA-PEs are oxidized to toxic phospholipid peroxidation products with the participation of lipoxygenases (LOXs)^37^, ^38^. In the non-enzymatic autoxidation reaction, the bisallylic hydrogen atom between two carbon-carbon double bonds can be removed from the polyunsaturated fatty acyl groups of phospholipids (PUFA-PLs) to form carbon-centered phospholipid radicals (PL·). Whereafter, the PL· can react with the oxygen molecules to generate the phospholipid peroxyl radical (PLOO·), which combines with the hydrogen atom removed from PUFA-PLs to yield the phospholipid hydroperoxide (PLOOH)^39^. With the accumulation of PLOOHs, PLOOHs and lipid radicals continue to react with PUFA-PLs, repeat the dehydrogenation and oxygenation process, intensifying the generation of PLOOHs (Figure 1)^40^, ^41^. Although the mechanisms of enzymatic and non-enzymatic reactions are complex and poorly studied, it is certain that the accumulation of lipid peroxidation can release highly cytotoxic products, such as 4-hydroxynonenal and malondialdehyde which destroy cell membranes, cell proteins and DNA^42^. ACSL4 and LPCAT3 are both important drivers and molecular markers of ferroptosis. The sensitivity of GPX4-ACSL4 double knockout cells to ferroptosis is significantly reduced, and inhibition of ACSL4 by thiazolidinediones can improve tissue damage in the mouse models of ferroptosis^43^. In contrast, increased expression of ACSL4 can enhance ferroptosis (Figure 1)^44^, ^45^. Besides, the susceptibility of cells to ferroptosis can be reduced by inhibition of LOX through vitamin E and α-tocotrienol, suggesting that LOX may also be involved in the mediation of ferroptosis^46^.

### GPX4/GSH/System Xc^-^ in Ferroptosis

GPX4 plays an essential regulator in the occurrence of ferroptosis. As a phospholipid hydroperoxidase, GPX4 can catalyze lipid peroxidation products to generate corresponding non-toxic lipid alcohols, thus decreasing sensitivity to ferroptosis^47^. GPX4 decreases ROS production and preserves cells from lipid hydroperoxides. GPX4 reduction of lipid hydroperoxides also needs electrons commonly from GSH or certain thiols^48^. GSH is a key antioxidant, consisting of cysteine, glycine and glutamate, which directly affects the activity of GPX4. The biosynthesis of GSH needs intracellular synthesis of cysteine, which requires the involvement of cystine-glutamate transporter receptors (System Xc^-^). System Xc^-^ is the sodium-dependent cystine/glutamate antiporter in the lipid bilayer, by which cells absorb extracellular cystine and output glutamate^49^. After cystine enters the cell, it is reduced to cysteine and then synthesized into GSH together with glutamate and glycine under the catalysis of glutathione synthetase and glutamate-cysteine ligase^50^. Finally, GPX4 oxidizes reduced GSH to oxidized GSH and reduces the cytotoxic lipid hydroperoxides to the corresponding lipid alcohols (Figure 1). The knockout of GPX4 or Ferroptosis suppressor protein 1 (FSP1) can aggravate ferroptosis^51^, ^52^. The light chain solute carrier family 7 member 11 (SLC7A11) of System Xc^-^ is its active subunit. Downregulation of SLC7A11 via erastin, AMPK or ATF3 can suppress ferroptosis (Figure 1) 16, 53, 54.

### FSP1/CoQ10/NAD(P)H in Ferroptosis

The FSP1/CoQ10/NAD(P)H is an endogenous antioxidant protection system that parallels to the GSH/GPX4, and inhibits ferroptosis by counteracting oxidative stress^55^. As a member of the quinone oxidoreductase family, FSP1 can be used to prevent ferroptosis caused by GPX4 deficiency. Bersuker et al. discovered ferroptosis resistance was positively associated with FSP1 expression levels in hundreds of cancer cell types, and FSP1 could inhibit ferroptosis both in cultured lung cancer cells and cancer xenografts in mice^52^. As a new and potent inhibitor of ferroptosis, N-terminal hydrophobic sequence myristoylation of FSP1 facilitates its recruitment to the plasma membrane, where FSP1 acts as an oxidoreductase to reduce NADH-dependent coenzyme Q10 (CoQ10) to coenzyme QH2 (CoQH2), which functions as a lipophilic radical-trapping antioxidant, thereby inhibiting lipid peroxidation (Figure 1) 56. Moreover, CoQ10 plays a key role in the electron transport chain of inner mitochondrial membrane, and the reduction of CoQ10 can directly affect ROS generation and suppress ferroptosis. Therefore, FSP1 inhibitors can induce ferroptosis, which may afford an efficient strategy for cancer therapy by making cancer cells more sensitive to chemotherapy drugs that induce ferroptosis.

### P53 in Ferroptosis

P53 is a typical tumor suppressor that plays a crucial part in cell cycle arrest, apoptosis, aging and tumorigenesis^57^. Multiple lines of evidence have suggested that P53 may play a bidirectional role in different cellular ferroptosis conditions: promotion or inhibition. Recent studies have found that P53 significantly reduced the SLC7A11 expression levels in specific cancer cells (Figure 1). Reduced expression of SLC7A11 inhibits System X_C_^-^, leading to reduced intracellular cysteine production, which ultimately inhibited the GSH/GPX4 axis, allowing the accumulation of lipid peroxidation products, thus promoting ferroptosis^58^. Furthermore, Jiang et al. induced P53-silenced H1299 cells with ROS did not have a large effect on the cell activity, however, approximately 90% of H1299 cells underwent ferroptosis after induction of normal H1299 cells with ROS^59^. In addition, P53 can mediate ferroptosis through enhancing the expression of spermine N1-acetyltransferase 1 and glutaminase 2, which have recently been identified as transcriptional targets of P53 in certain cells (such as MCF7, H1299, A375) (Figure 1) 60, 61. Interestingly, P53 can also inhibit ferroptosis in some cells. For instance, Amy et al. reported that HT-1080 fibroblasts treated with the P53 inducer Nutlin3 exhibited a lower susceptibility to ferroptosis if they were induced with erastin-2. Kang et al. concluded that P53 could restrain ferroptosis by directly depressing dipeptidyl peptidase 4 (DPP4) activity or mediating cyclin dependent kinase inhibitor 1A (CDKN1A/p21) expression (Figure 1)^61^.

## Pathogenesis of DC

Cardiac insulin resistance, mitochondrial dysfunction, impaired calcium handling, oxidative stress, AGEs, and inflammation may be the main pathogenesis of DC.

Physiologically, insulin signaling maintains homeostasis in the cardiomyocyte intracellular environment by regulating cardiac physiological activities such as protein synthesis, cell state, and substrate usage. After binding of insulin to the insulin receptor on the surface of cardiomyocytes, the insulin signaling/docking molecules insulin receptor substrate-1/2 and downstream PI3K /Akt are activated to transfer glucose transporter type 4 (GLUT4) to the cell membrane, mediating glucose uptake^62^. Decreased PI3K/Akt signaling as well as decreased GLUT4 expression have been found in myocardial biopsies from DM patients, confirming the presence of cardiac insulin resistance^63^. Cardiac insulin resistance impacts cardiac metabolism, increases ROS production, and leads to mitochondrial dysfunction.

Mitochondrial dysfunction can cause a shift in mitochondrial metabolic substrates in cardiomyocytes. Normally, approximately 95% of ATP in cardiomyocytes is supplied by mitochondria, with an additional 5% supplied by the citric acid cycle^64^, ^65^. However, in DM, mitochondrial dysfunction reduces the ability of cardiomyocytes to utilize glucose as a primary energy source, thereby turning to FFAs^65^. This shift in metabolic substrates cause impairment of oxidative phosphorylation as well as the leakage of mitochondrial protons, leading to increased ROS production. In addition, cardiomyocyte glucose overload promotes AGEs formation, which exacerbate myocardial fibrosis and decrease myocardial compliance^66^, ^67^. AGEs also bind to its receptors, which mediates inflammatory reaction, promotes ROS production and promotes connective tissue production and fibrosis^62^.

Cardiomyocytes from DC patients have impaired calcium handling. During Cardiac excitation-contraction coupling, Ca^2+^ enters cardiomyocytes via voltage-gated L-type Ca^2+^ channels after depolarization of the cell membrane, and mediates Ca^2+^ release from the sarcoplasmic reticulum via the ryanodine receptors. Binding of Ca^2+^ to troponin C mediates myofibril contraction^68^, ^69^. During cardiac diastole, the sarcoplasmic reticulum reuptakes most intracellular Ca^2+^, and a few Ca^2+^ are pumped out of the cell through the Na^+^/Ca^2+^ exchangers and plasma membrane Ca^2+^ pumps^68^, ^69^. However, in DC, the Ca^2+^ handling by these transporters is impaired, resulting in prolonged intracellular action potential duration, increased resting Ca^2+^, and slowed Ca^2+^ transients, leading to impaired myocardial systolic and diastolic function^70^, ^71^. Similar changes have been seen in type 1 diabetes mellitus (T1DM) rodent models and type 2 diabetes mellitus (T2DM) mouse models^71^, ^72^.

The heart has limited antioxidant capacity, and when mitochondrial ROS production exceeds the endogenous scavenging capacity, it can lead to oxidative stress and inflammation response, aggravating myocardial fibrosis and hypertrophy. Normally, the proton electrochemical gradient of the mitochondrial respiratory chain is mostly used for ATP synthesis, and ROS are metabolic by-products of complexes I and III in the mitochondrial respiratory chain^62^. However, in DC, the nicotinamide adenine dinucleotides and flavin adenine dinucleotides lead to hyperpolarization of the inner mitochondrial membrane, which affects electron transport in complex III and ultimately produces excess ROS^73^. Other sources of ROS in cardiomyocytes in DC are increased activity of oxidation enzymes such as xanthine oxidase and microsomal cytochrome P-450 enzymes^74^. Excessive ROS can activate multiple key redox-related enzymes, further exacerbating the impairment of cardiac function and structure^75^.

The chronic inflammatory state caused by DM is associated with DC. A maladaptive proinflammatory response induced by cytokines is involved in cardiac oxidative stress, myocardial remodeling, and myocardial fibrosis^62^. The nucleotide-binding oligomerization domain-like receptor family, pyrin domain-containing 3 (NLRP3) is a molecular marker of DC, which can be activated under conditions of hyperglycemia, high FFA levels, and impaired insulin signaling^76^. Activated NLRP3 segregates and oligomerizes from molecular chaperones, activating caspase-1, which in turn induces the production of interleukin-1β and interleukin-18, contributing to local tissue inflammation. The NF-κB positive feedback loop can further mediate ROS-induced activation of NLRP3 inflammasome effectors caspase-1 and interleukin-1β^76^. Silencing NLRP3 can improve cardiac inflammation, myocardial fibrosis, and cardiac function in DM rats^77^.

## Roles of Ferroptosis in DC

### Ferroptosis caused by retinol metabolism disorders

**Figure 2:**
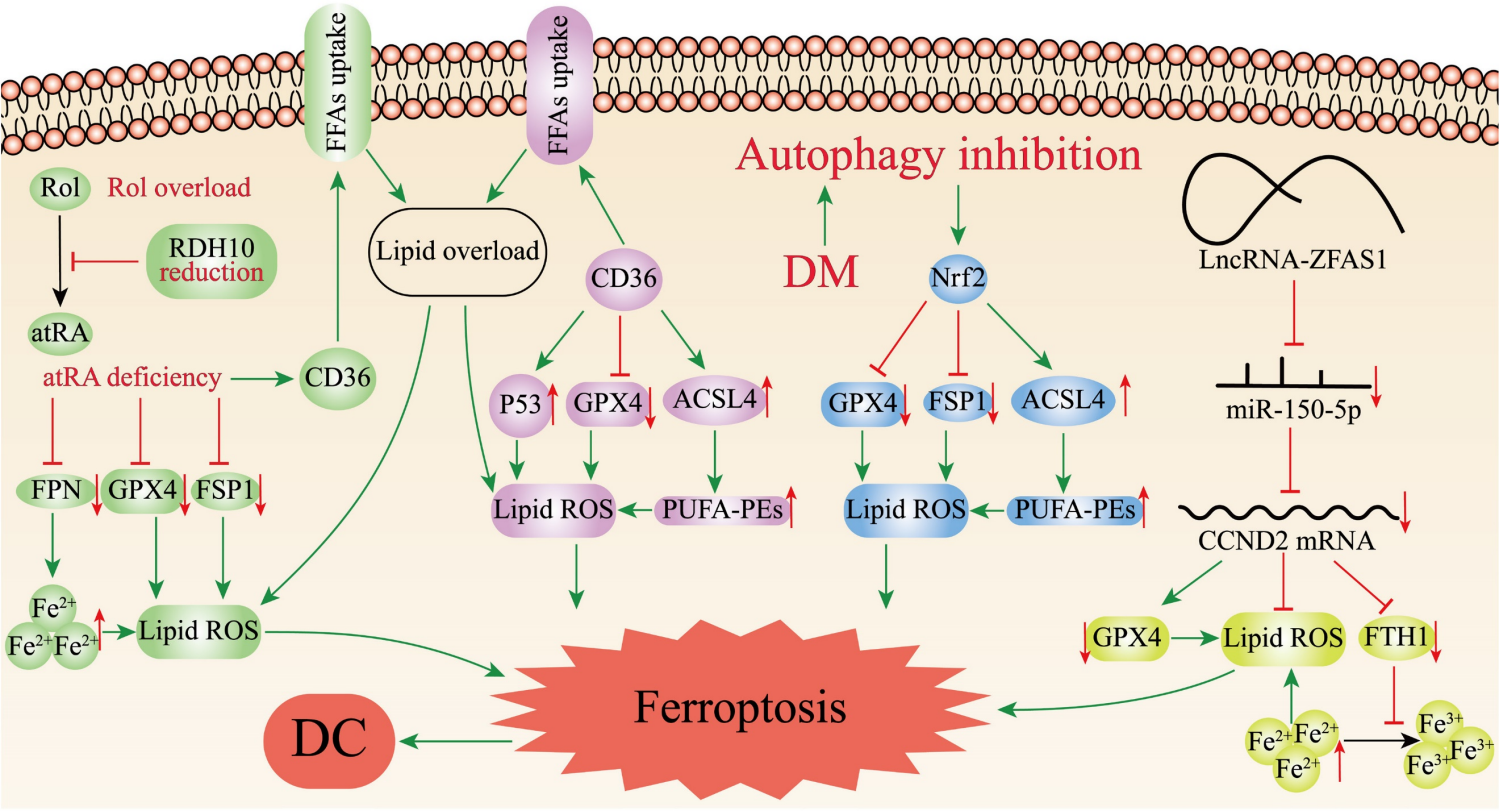
**Ferroptosis-related mechanisms in DC.** The figure demonstrates the pathways mediating ferroptosis in DC cardiomyocytes. Disorders of cardiac retinol metabolism due to reduced RDH10 can lead to atRA deficiency, which can mediate ferroptosis through depressing GPX4, FSP1 and FPN1; CD36 can promote ferroptosis by up-regulating ACSL4 or P53, down-regulating Gpx4, and enhancing lipid deposition. Inhibition of myocardial autophagy activates Nrf2, which increases ACSL4 expression as well as inhibits GPX4 and FSP1, and mediates ferroptosis; ZFAS1 is highly expressed in DC and can bind miR-150-5p to downregulate CCND2 expression, thereby promoting ferroptosis.

Retinol (vitamin A, Rol) is a key micronutrient for the regulation of cell differentiation, cell metabolism and stem cell function^78^. During retinol metabolism, Rol acts as the metabolic substrate to yield all-trans retinoic acid (atRA) through dehydrogenation, which binds to ligands to exert biological effects. Retinol dehydrogenase 10 (RDH10) is the rate-limiting enzyme in this process and is essential in the transition of Rol to atRA^78^. Wu et al.^79^ found that retinol metabolism disorders featured by Rol overload, atRA shortage, and decreased ligands in the hearts of T2DM mice. Silencing RDH10 in Neonatal mouse primary cardiomyocytes (NMPC) could induce lipid deposition by reducing atRA to increase CD36 expression, and lipid deposition could be ameliorated by atRA but not Rol. CD36 is a crucial factor in the abnormal uptake of free fatty acids (FFAs) in the heart^80^. Subsequently, they observed iron overload in the hearts of T2DM mice and RDH10-knockout mice on a high iron diet, which could be significantly inhibited by RDH10 and atRA. Changes in cardiac retinol metabolism also affected the expression of FPN1. What's more, the study detected increased levels of 4-hydroxynonenal and malondialdehyde in the hearts of RDH10-knockout mice, while using the ferroptosis inhibitor ferrostatin-1 could suppress the accumulation of 4-hydroxynonenal and ameliorate HF, suggesting ferroptosis was mainly caused by lipid peroxidation. The study further detected ferroptosis-related regulatory molecules, and showed that cardiac RDH10 deficiency in RDH10-knockout mice decreased the expression of GPX4 and FSP1, while atRA but not Rol in NMPC restored the silencing RDH10-induced GPX4 and FSP1 expression. The expression of GPX4 and FSP1 in T2DM mice was restored, and lipid peroxidation was significantly reduced by the supplementation of RDH10 and atRA. The above evidences demonstrated that cardiac retinol metabolism disorders due to reduced RDH10 could lead to atRA deficiency, which could mediate ferroptosis through decreasing the expression of GPX4, FSP1 and FPN1, ultimately leading to myocardial remodeling (Figure 2).

### Ferroptosis Mediated by CD36

CD36 is a kind of multifunctional receptor that plays an important part in lipid metabolism, inflammatory response, reprogramming of energy metabolism and angiogenesis, and involved in the transport of various lipids^81^. Recent studies have reported that CD36 could promote fatty acids uptake by CD8+ T cells and induce lipid peroxidation and ferroptosis in the tumor microenvironment^82^. CD36 is highly expressed in cardiomyocytes of DC. Li et al.^83^ confirmed that CD36 could up-regulate ACSL4 and P53, down-regulate the mRNA and protein expression of Gpx4, promote ROS production, enhance intracellular lipid deposition, and lead to ferroptosis in DC cardiomyocytes, resulting in myocardial injury and cardiac dysfunction *in vivo* and *in vitro* experiments (Figure 2). At the same time, they found that Astragaloside IV (AS-IV) could counteract CD36-induced lipid deposition, ROS production, and downregulate P53, ACSL4 and upregulate GPX4 in DC rat hearts, restore left ventricular ejection fraction and lessen myocardial inflammatory infiltration and interstitial fibrosis. AS-IV, one of the mainly active ingredients isolated from *Astragalus membranaceus*, has anti-inflammatory and antioxidant functions, and is expected to be an effective drug for the treatment of DC^84^ (Table 1).

### Ferroptosis Mediated by Nrf2

The transcription factor nuclear factor-erythroid factor 2-related factor 2 (Nrf2) regulates the expression of various genes, such as antioxidant genes, scavenger receptors, transporter proteins, autophagic degradation and phase II detoxification enzymes. Nrf2 is an essential anti-oxidative stress regulator *in vivo*, which is expected to be an effective medicinal target for the therapy of oxidative stress-related diseases^85^. Several recent studies have revealed Nrf2 is associated with ferroptosis in DC cardiomyocytes, myocardial injury and therapy. Zang et al.^86^ first determined that T1DM could suppress myocardial autophagy and induce myocardial cell death by studying the time course of myocardial autophagy flux in the mouse models of T1DM and CR knockout of autophagy-related 5 gene (CR-Atg5KO) in myocardial cells. Secondly, by knocking out Atg5 and Nrf2 genes in the hearts of T1DM mice, it was discovered that CR-Atg5KO aggravated myocardial remodeling after Nrf2 knockout (Nrf2KO), which further established that T1DM induced Nrf2 activation through suppressing myocardial autophagy, thus leading to cardiac injury and dysfunction. Then, measurement of iron and lipid peroxidation products in the hearts of T1DM mice revealed increased deposition of the cardiomyocyte iron and lipid peroxidation after 9 months of T1DM, which could be reversed by Nrf2KO. Subsequently, ferroptosis was induced in H9C2 cells with erastin to verify the role of Nrf2, and it was found that erastin induced ferroptosis by deactivating GPX4 and decreasing the expression of FSP1 while upregulating ACSL4 related to Nrf2 activation^87^. Ultimately, the study determined that DM could activate Nrf2 by inhibiting myocardial autophagy, and Nrf2 mediated iron overload and lipid peroxidation in DC, which in turn promoted ferroptosis in cardiomyocytes (Figure 2).

### Ferroptosis Mediated by ZFAS1

Long non-coding RNAs (lncRNAs) are non-coding RNAs longer than 200 nucleotides, which play a significant role in various biological processes, such as cell differentiation, cell metabolism, apoptosis^88^. Zinc finger antisense 1 (ZFAS1) is a novel lncRNA related to the development of multiple diseases including pulmonary fibrosis, myocardial infarction, and atherosclerosis^89^-^91^. Ni et al.^92^ initially found upregulation of ZFAS1 expression, downregulation of miR-150-5p expression and ferroptosis in high glucose (HG)-treated cardiomyocytes and DC mouse models. Given the precedent of lncRNAs being extensively involved in pathophysiological processes as competing endogenous RNAs (ceRNAs) absorbing biomolecules, and the fact that miR-150-5p was proven to be a predictor of HF^93^-^95^, Ni et al. continued to identify the specific mechanism of ZFAS1 and miR-150-5p in DC. They inhibited ZFAS1 in DC- and HG-treated cardiomyocytes, and found that upregulation of miR-150-5p as well as suppression of ferroptosis, clarifying that ZFAS1 and miR-150-5p were associated with ferroptosis. Then, ZFAS1 and miR-150-5p were respectively overexpressed in DC-and HG-treated cardiomyocytes, finding that miR-150-5p overexpression consistent with the restraining effect of ZFAS1 could elevate the expression levels of FTH1 and GPX4 to alleviate ferroptosis, while ZFAS1 overexpression could reverse the positive effect of miR-150-5p. Interestingly, they found that another molecule, Cyclin D2 (CCND2), had a consistent expression trend with miR-150-5p. Besides, overexpression of CCND2 showed a significant inhibitory effect on ferroptosis, resembling the inhibitory effect of ZFAS1. On the contrary, these effects would be counteracted when ZFAS1 stimulation was simultaneously enhanced. Ultimately, the study concluded that ZFAS1, as ceRNA bound to miR-150-5p, decreased CCND2 expression levels contributing to ferroptosis and DC development in cardiomyocytes (Figure 2)^92^.

## Discussion

In this review, we take ferroptosis as the starting point and summarize the mechanisms of ferroptosis in DC, and finally clarify the important position of ferroptosis in the pathogenesis of DC. Retinol metabolism disorders, CD36, Nrf2, and ZFAS1 can be involved in DC development through their respective pathways mediating ferroptosis.

Retinol metabolism has not been given much attention in the cardiovascular field, but its link with ferroptosis has been demonstrated in liver injury in mice^96^. The finding that cardiac retinol metabolism disorders mediate ferroptosis to promote DC is of great significance. Supplementation with atRA, RDH10, and avoidance of Rol can alleviate ferroptosis and ameliorate myocardial injury by correcting retinol metabolism disorders, which can hopefully be termed as potential targets for DC therapy (Table 1). Notably, the finding is not limited to mouse experiments. Retinol metabolism disorders were also found in forensically collected heart sections from patients with T2DM^79^. However, samples from forensic sources lack much patient information, greatly increasing the limitations of human validation. Fortunately, previous study has confirmed the presence of increased Rol and decreased atRA in the hearts of patients with HF filling the gap in human validation^97^. In addition, the finding is limited to T2DM, whether the same changes exist in T1DM is unknown. As a metabolic substrate for retinol metabolism, Rol has been paradoxically studied in cardiovascular diseases. A study tested whether β-carotene and Rol could prevent cancer showed that the supplements led to a 26% increased risk of cardiovascular death in the intervention group^98^. In contrast, another case-control study found that increased plasma Rol concentrations were associated with reduced coronary artery disease^99^. Obviously, follow-up studies should clearly elucidate the specific relationship between Rol and cardiovascular diseases.

In the cardiovascular system, CD36 is not only expressed in cardiomyocytes, but also present in other cells, such as endothelial cells. CD36 is a crucial factor in the abnormal uptake of FFAs by the heart^80^. It has been found that increased expression of CD36 in DM db/db mice accompanied by increased oxidation of FFAs, significant cardiac systolic dysfunction, elevated left ventricular diastolic pressure, and reduced cardiac output^100^. Whereas, knockout of CD36 reduced oxidative stress. The finding that CD36 mediates ferroptosis in cardiomyocytes certainly further explains this phenomenon. Nevertheless, in DC, CD36 may be an important intermediate in mediating the onset of ferroptosis, participating in a variety of ferroptosis response pathways. Furthermore, given the efficacy of AS-IV in treating DC, further studies could concentrate on CD36-targeted therapy in DC patients, as well as digging into other possible mechanisms of ferroptosis.

The roles of Nrf2 in the DM hearts have been controversial. For example, the cardioprotective effect of Nrf2 was observed in both T1DM and T2DM mice, while a deleterious axis of Nrf2-CD36-lipotoxicity was also found in T1DM fibroblast growth factor21 KO mice^101^-^103^. This review uncovers Nrf2-mediated ferroptosis in cardiomyocytes, whereas another study showed that a large number of natural compounds, such as radical acid sulfur and resveratrol could enable Nrf2-mediated cardio-protection in DM animal models^6^. Zang et al. found that Nrf2 regulated the expression of genes related to iron and lipid metabolism as well as redox levels in the heart to sustain the dynamic balance of metabolism and redox in DC when myocardial autophagy was functioning well. Nonetheless, when myocardial autophagy was impaired, the metabolic and redox homeostasis controlled by Nrf2 was disrupted and Nrf2 then mediated cardiomyocyte ferroptosis^86^. This is consistent with previous reports that activation of Nrf2 was cardioprotective when myocardial autophagy normal. It not only helps to explain the controversy about Nrf2 in the DM hearts, but also provides new insights into the pathogenesis of DC. Notably, Wei et al.^104^ and Wu et al.^105^ also demonstrated the occurrence of Nrf2-mediated ferroptosis in DC cardiomyocytes, and they suggested that curcumin or 6-Gingerol could treat DC via this pathway, providing the possibility of targeting Nrf2 to treat DC(Table 1). Moreover, the above findings are limited to T1DM mouse models and Rat H9C2 cells, lacking T2DM and human experimental validation.

In the last decade, the role of lncRNAs has been found to be primarily associated with various cardiovascular diseases, especially myocardial infarction^95^. Yet, the role of lncRNAs in the pathogenesis of DC remains unclear. The finding that lncRNA-ZFAS1 mediates ferroptosis in DC cardiomyocytes fills this gap. It is also consistent with reports from other related studies, such as the GSE26887 dataset from the GEO database, which showed that lncRNA-ZFAS1 was significantly upregulated in DM patients with comorbid HF compared with DM patients, whereas downregulation of ZFAS1 led to a protective effect on cardiomyocytes during myocardial infarction. LncRNAs have been proven to participate in a variety of pathophysiological processes as CeRNAs sponging biomolecules such as microRNAs and proteins^95^. Previous clinical study has shown that miR150-5p levels were markedly decreased in patients with HF. The miR-150-5p gene attenuates apoptosis in sepsis-induced myocardial depression, and slows down the progression of myocardial fibrosis and protects cardiomyocytes from hypoxic injury under the regulation of lncRNA FOXD3-AS1^106^, ^107^. Here, the finding that ZFAS1 can act as a ceRNA to sponge miR-150-5P thus mediating ferroptosis, which provides a new perspective on DC pathogenesis. More importantly, inhibition of ZFAS1 inhibits cardiomyocyte ferroptosis and slows DC progression (Table 1). Targeting ZFAS1 makes it possible to further develop novel methods for DC treatment.

Undoubtedly, research on the mechanisms of ferroptosis in DC is still in its infancy. For example, iron is an essential element of ferroptosis, and iron metabolism is a necessary process of ferroptosis, but the mechanism of iron metabolism in DC is still unknown. When intracellular iron is overloaded, Fe^2+^ not only causes a strong oxidative stress response, but also acts as a cofactor to enhance the activities of various metabolic enzymes, promotes the production of ROS, and induces ferroptosis. In DM patients, ferritin levels have elevated in the body and therefore cells are more susceptible to iron overload. Since pancreatic βcells lack a powerful antioxidant mechanism, iron overload can directly affect insulin secretion and insulin sensitivity, exacerbating DM. In addition, oxidative stress is the underlying pathogenesis of DM, hyperglycemia upregulates chronic inflammatory markers and increases ROS production, and iron overload undoubtedly further exacerbates oxidative stress. The mechanisms of specific iron metabolism in DC still needs to be further explored. The end stage of DC is HF. Iron deficiency is particularly common in patients with HF, accounting for 47% to 68% of HF patients. Myocardial iron deficiency promotes and exacerbates myocardial ischemia, leading to myocardial remodeling. Iron supplementation replenishes myocardial iron stores and appears to mitigate the harmful consequences of myocardial iron deficiency^108^. Randomized trials of intravenous iron supplementation in patients with HF have shown that intravenous iron supplementation demonstrates significant prognostic benefits in patients with serum transferrin saturation <20% and may have deleterious consequences in patients with transferrin saturation >24%^109^. It is worth noting that serum ferritin levels are elevated in DM patients and that cardiomyocyte iron overload is an important pathogenetic mechanism of DC, so DC patients do not seem to be iron deficient. With the progression of DC to the stage of HF, how the body's iron metabolism changes remain unknown, and how to balance the iron content of cardiomyocytes is still facing a huge challenge. Therefore, researches on DC still have a long way to go.

## Figures and Tables

**Table 1:** Potential targeted drugs or interventions targeting ferroptosis for DC.

Targeted drugs or interventions	Mechanism	Effects on the heart	References
Supplementing atRA and RDH10, avoiding Rol	Correction of retinol metabolism disorders	Ameliorating myocardial injury	79
AS-IV	Downregulating P53 and ACSL4, upregulating GPX4, ameliorating lipid deposition and ROS production	Restoring left ventricular ejection fraction, reduction of myocardial inflammatory infiltration and interstitial fibrosis	83
Curcumin, 6-Gingerol	Inhibiting ACSL4, increased expression of Gpx4 and HO-1, decreased secretion of inflammatory cytokines	Amelioration of cardiac hypertrophy and fibrosis	104, 105
Inhibiting ZFAS1	Inhibiting ferroptosis by sponging miR-150-5p to activate CCND2	Alleviating myocardial fibrosis, attenuating DC progression	92

ACSL4, Acyl-CoA synthetase long-chain family member 4; AS-IV, Astragaloside IV; atRA, All-trans retinoic acid; CCND2, Cyclin D2; DC, Diabetic cardiomyopathy; ACSL4, Acyl-CoA synthetase long-chain family member 4; GPX4, Glutathione peroxidase 4; HO-1, Heme Oxygenase-1; RDH10, Retinol dehydrogenase 10; Rol, Retinol; ROS, Reactive oxygen species; ZFAS1, Zinc finger antisense 1.
